# Special Issue Dedicated to Dr. Gambhir and Dr. Kircher

**DOI:** 10.7150/ntno.68107

**Published:** 2022-01-01

**Authors:** Jonathan F Lovell

**Affiliations:** Editor in chief, Nanotheranositcs; Associate Professor of Biomedical Engineering, State University of New York at Buffalo, Buffalo, New York, USA

The field of Nanotheranostics, the namesake of this journal, lost two foundational pillars last year. It is difficult to accept that Dr. Sam Gambhir and Dr. Moritz Kircher both passed away much too soon. Two individuals who dedicated their careers to, what at the time they started, was radical new research fusing molecular imaging, therapy and nanoscale design. Both were founding editorial board members of Nanotheranostics. Dr. Kircher was the editor in chief of Nanotheranostics at the time of his passing. These physician scientists were motivated to translate this type of innovative basic research into their clinics in order to benefit humankind.

Stanford Radiology, where both spent significant time, represents an intellectual headquarter and origin for much of the theranostics field. Indeed, it is difficult to imagine research on the topic not being strongly influenced by work carried out by scientists who spent time there. It should be sadly noted that in 2018, Stanford radiologist Dr. ‪Jürgen Willmann was lost in a car accident at age 45. The string of untimely departures is a terrible coincidence that, besides personal loss for friends and family, significantly sets back progress to nanotheranostics. These physician scientists were pioneering a brand of human health research that was multidisciplinary, clinically-minded, and highly innovative. It is unclear when their roles can be filled in the future by other researchers as driven and talented as they were.

It was therefore with mixed emotions that I assumed the role of Editor-in-Chief of Nanotheranostics last year. I will try to follow in the editorial footsteps of Dr. Kircher. It should also be said that despite the setbacks, there are tremendously exciting developments with respect to the current state of the field, that provide grounds to be optimistic about the future. Nanotheranostics, as a journal, will continue to publish cutting edge research in the field.

This special memorial issue aims to honor the legacy of Dr. Gambhir and Dr. Kircher. It features articles contributed posthumously by both Dr. Gambhir, Dr. Kircher, and also the late Dr. Mike Detty, who made contributions to the field of Raman spectroscopy dyes. Enormous thanks go to all authors, and the corresponding contributors to this issue, including Dr. Stefan Harsen, Dr. Ramasamy Paulmurugan, Dr. Jesse V. Jokerst, Dr. Chrysafis Andreou, Dr. Jeesu Kim, Dr. Peng Huang, Dr. Xiaoyuan Chen, Dr. Carina Mari Aparici and Dr. Natesh Parusharama.

